# Influence of regional environmental variables on the radiative forcing of atmospheric microplastics

**DOI:** 10.1016/j.eehl.2024.11.002

**Published:** 2024-12-09

**Authors:** Hanling Yang, Yining Xue, Xiaoyu Sha, Jintao Yang, Xinling Wang, Balt Suvdantsetseg, Keisuke Kuroda, Jian Pu, Lei Wang

**Affiliations:** a MOE Key Laboratory of Pollution Processes and Environmental Criteria/Tianjin Key Laboratory of Environmental Remediation and Pollution Control, College of Environmental Science and Engineering, Nankai University, Tianjin 300350, China; b Department of Administration and International Cooperation, Mongolian Academy of Sciences, Ulaanbaatar, Mongolia; c Department of Environmental and Civil Engineering, Toyama Prefectural University, Toyama 939-0398, Japan; d Institute for the Advanced Study of Sustainability, United Nations University, Tokyo 150-8925, Japan; e Institute for Future Initiatives, The University of Tokyo, Tokyo 113-0033, Japan

**Keywords:** Atmospheric microplastics, Radiative forcing, Environmental variables, Radiative effects, Surface albedo

## Abstract

Atmospheric microplastics (AMPs) can absorb and scatter radiation, which can be quantified by radiative forcing. Although the radiative forcing of AMPs is commonly positive at the global scale, regional environmental variables affect the radiative forcing of aerosols, potentially reversing its directions and cause opposite radiative impacts in the atmosphere. In this study, the total suspended particles were collected within one year in Tianjin, China, and the monthly average concentration of AMPs of 200.0–463.9 items/m^3^ was detected. Accordingly, the direct radiative forcing (DRF) of AMPs was calculated as −0.03 to 0.03 W/m^2^ at the top of the atmosphere and −0.09 to 0.72 W/m^2^ at the Earth's surface, respectively. The surface albedo significantly affects the direction of the DRF of AMPs. A low surface albedo leads to a cooling effect of AMPs in the atmosphere in Tianjin, while a high surface albedo causes a warming effect in the atmosphere. The DRF calculated under different surface albedo indicates that the potential impact of AMPs on atmospheric temperature is relatively complex. The temperature changes caused by AMPs in grassland and bare soil areas may be opposite to those in areas covered by snow and ice.

## Highlights

• AMPs pollution was monitored for a year in one of the biggest cities in northern China.
• In Tianjin, the DRF of AMPs at TOA and ES were −0.03 to 0.03 W/m^2^ and -0.09 to 0.72 W/m^2^, respectively.
• Surface albedo showed a great influence on the direction of the DRF of AMPs.
• AMPs over ocean, forest, snowfield and glacier tend to cause a cooling effect, while AMPs over grassland and bare soil tend to cause a warming effect.

## Graphical abstract

**Figure:**
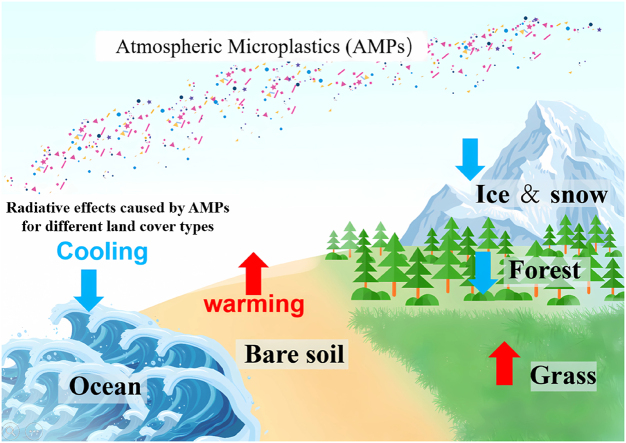

## 1 Introduction

Atmospheric aerosols, such as black carbon, sulfate, and nitrate aerosols, may affect climate by their direct radiative effects of absorbing and scattering radiation [[1], [2], [3]]. These effects can be quantified with the radiative forcing metric. As a large amount of plastic waste has accumulated at the Earth's surface (ES) [4], some microplastics (MPs) can enter the atmosphere and be transported to remote areas [5,6]. Plastics have the ability to absorb and scatter radiation [[7], [8], [9], [10]]. Considering that atmospheric microplastics (AMPs) can exist for a longer time in the atmospheric environment due to their chemical stability, the radiative forcing caused by AMPs should not be ignored [11]. In addition, the rapid growth of plastic production over the past few decades [12,13] may result in more MPs entering the atmosphere. Therefore, it is expected that the concentration of AMPs and their radiative effects will continue to increase.

The radiative forcing of aerosols is obtained by summing their longwave and shortwave band effects. The net radiative forcing at the top of the atmosphere (TOA) can be used to estimate climate effects caused by AMPs, which was estimated as −0.036 to 0.044 W/m^2^ in a previous study [11], assuming an AMPs concentration at ES of 100 items/m^3^. In addition, the net radiative forcing of AMPs at ES, which has been commonly used to estimate the change of surface energy budget induced by the aerosols [14], has not yet received attention.

Unlike other aerosols that mainly cause radiative effects in the shortwave band [[1], [2], [3]], AMPs cause radiative effects in both the shortwave and longwave bands [11]. Similar to sulfate and nitrate aerosols [3], AMPs scatter the shortwave radiation, thus causing a negative radiative forcing [11]. Meanwhile, AMPs absorb the longwave radiation [11] and lead to a radiative effect similar to that of greenhouse gases, resulting in a positive radiative forcing [11,15]. The opposite radiative forcings of AMPs in longwave and shortwave bands lead to uncertainty in the direction of net radiative forcing [11], which determines the cooling or warming effects of AMPs at TOA and ES. Environmental variables, such as ozone, water vapor, and surface albedo, may induce different degrees of changes in the shortwave and longwave radiative forcing of AMPs, thus affecting the direction of net radiative forcing [1]. However, research on the influences of environmental variables on the radiative forcing of AMPs is limited.

To evaluate the radiative forcings of AMPs at both TOA and ES and to discuss the influence of environmental variables on their magnitude and direction, a year-long monitoring of AMP concentration was conducted in Tianjin, one of the largest cities in northern China. Environmental variables, including water vapor, ozone, and surface albedo [1], were taken into account in the calculation of the direct radiative forcing (DRF, excluding aerosol–cloud interactions) of AMPs. In addition, at the lowest and highest levels of AMP concentration in Tianjin, the DRF of AMPs at TOA and ES was assessed at different surface albedos.

## 2 Materials and methods

### 2.1 Sample collection

Tianjin was selected as the study location. Atmospheric suspended particulate matter was collected twice a month from September 2021 to August 2022 on the roof of a building (3 m above the ground) located in the urban area (Fig. S1). Samples were collected on clear weather by a middle-flow total suspended particulate sampler (TH-150 AII, Tianhong, Wuhan) with glass microfiber filters (Whatman GF/A, 90 MM, 1.6 μm) for 24 h at an intake flow rate of 100 L/min. Details of the preservation and pre-treatment of samples were described in Text S1.

Meteorological data of Tianjin during the sampling period, including wind speed, wind direction, rainfall, pressure, relative humidity, and snowfall amount (Table S1), were obtained from the European Centre for Medium-Range Weather Forecasts (https://www.ecmwf.int/).

### 2.2 Microplastics detected by laser direct infrared image (LDIR)

An LDIR imaging system (Agilent 8700, Agilent Technologies Inc., Santa Clara, CA) equipped with Agilent Clarity software and a library of Microplastics Starter 1.0.1 was used to identify the AMPs, with a detection limit of 10 μm. The standard spectra (975–1800 cm^−1^) were derived from the determination of the LDIR imaging system to standards. Due to their small particle size, MPs in dust and atmospheric samples may be more severely affected by surface aging. Thus, a matching degree exceeding 65%, which was used to identify MPs in the indoor dust [16,17], was selected in this study.

### 2.3 Calculation of the DRF of AMPs

For a given atmospheric height, aerosol DRF can be calculated as the difference in radiative fluxes from an aerosol-free atmosphere to an aerosol-laden atmosphere. In this study, DRFs of AMPs at TOA and ES were calculated by the Santa Barbara Discrete Ordinate Radiative Transfer (SBDART) model [18], a widely used model in estimating the radiative forcing of various aerosols [1,[19], [20], [21], [22]]. Optical parameters of MPs used in SBDART include the spectral single scattering albedo (SSA), asymmetric factor (ASY), and aerosol optical depth (AOD).

Factors such as composition, color, and size distribution may influence the optical parameters of AMPs [23]. However, as different polymers exhibit similar refractive indexes [[7], [8], [9], [10],^24^], the impact of AMP composition on the radiative forcing is limited [11]. Determining the refractive index of pigmented plastics is challenging because of the complex composition of the pigments. Since the majority of AMPs identified in this study were found to be white and transparent (Text S2 and Fig. S2), the extra contribution of colored AMPs was ignored herein. Since the average size of AMPs of Tianjin (35 μm, Fig. S3) is similar to that observed in previous research (36 μm), values for SSA and ASY from a previous study [11] were adopted here as these variables are not affected by AMPs concentration or environmental variables. The AOD of AMPs can be obtained by following the steps below.

The vertical distribution of AMPs is poorly understood because most observations of AMPs were conducted on the ground [5,6]. Thus, the vertical distribution of AMPs in general was assumed to correspond with the air density at different altitudes [11], as follows,

(1) $${\left[\mathrm{MP}\right]}_{z}=N_{0}\times 0.3^{z/10}$$

where *z* is the height above the ground (km), *N*_*0*_ is the AMP (items/m^3^) concentration on ES, and [MP]_*z*_ is MP concentration (items/m^3^) in the atmosphere at altitude *z*. Since the SBDART requires the inputs of optical parameters for boundary layer aerosols in order to obtain the aerosol radiative forcing at TOA and ES, the variable *z* was set to 2 km in this study, representing the typical height of the boundary layer [18]. Currently, there are still no reliable quantitative methods for quantifying MPs in the size range of several micrometers. A previous research, based on AMP size distributions reported in other studies that excluded particles below 10 μm, proposed a gamma distribution that predicted the distribution of AMPs below 10 μm [11]. This indicates that AMPs between 0 and 10 μm accounted for approximately 25% of the total AMPs [11]. Since the average size of AMPs larger than 10 μm of Tianjin (35 μm, Fig. S3) is similar to that of the gamma distribution (36 μm), it is assumed that the portion of AMPs below 10 μm in this study constitutes 25%. Then, the average atmospheric column concentration of MPs (N_MPs_, items/m^3^) can be calculated as Eq. 2.

(2) NMPs=∫0zMPzdzz

After N_MPs_ was determined, the corresponding wavelength-dependent absorption coefficients (σ_abs_) and scattering coefficients (σ_sca_) of AMPs can be obtained by multiplying the σ_abs_ and σ_sca_ of the source data [11] by N_MPs_ (estimated in this study)/N_MPs_ (from source data), as σ_abs_ and σ_sca_ change linearly with N_MPs_. Then, the aerosol optical depth (AOD) of MPs, an important parameter characterizing the extinction properties of aerosols, can be calculated based on Eq. 3.

(3) $$\text{AOD}\;\left(\lambda \right)=\int_{0}^{z}\left[\sigma_{\text{abs}}\;\left(\lambda \right)+\sigma_{\text{sca}}\;\left(\lambda \right)\right]dz$$

where λ is the wavelength.

In addition to the optical parameters, background parameters, including columnar ozone, surface albedo, and columnar water vapor, were also used in the SBDART model (Table S2). By inputting the background parameters and spectral SSA, ASY, and AOD, the integrated DRF of AMPs in shortwave range (0.25–4 μm) and longwave range (4–40 μm) can be obtained by the SBDART model (The optical parameters of AMPs at different AMP concentrations can be seen in Table S3).

### 2.4 Statistical analysis and quality assurance

Statistical analyses, including Spearman's correlation and Mann–Whitney *U* test, were conducted in SPSS software (25.0, IBM, U.S.A.). Significance was considered at 0.05 alpha level. The detailed quality assurance for the AMP detection are described in Text S3.

## 3 Results and discussion

### 3.1 Concentration of atmospheric MPs in Tianjin

**Fig. 1:**
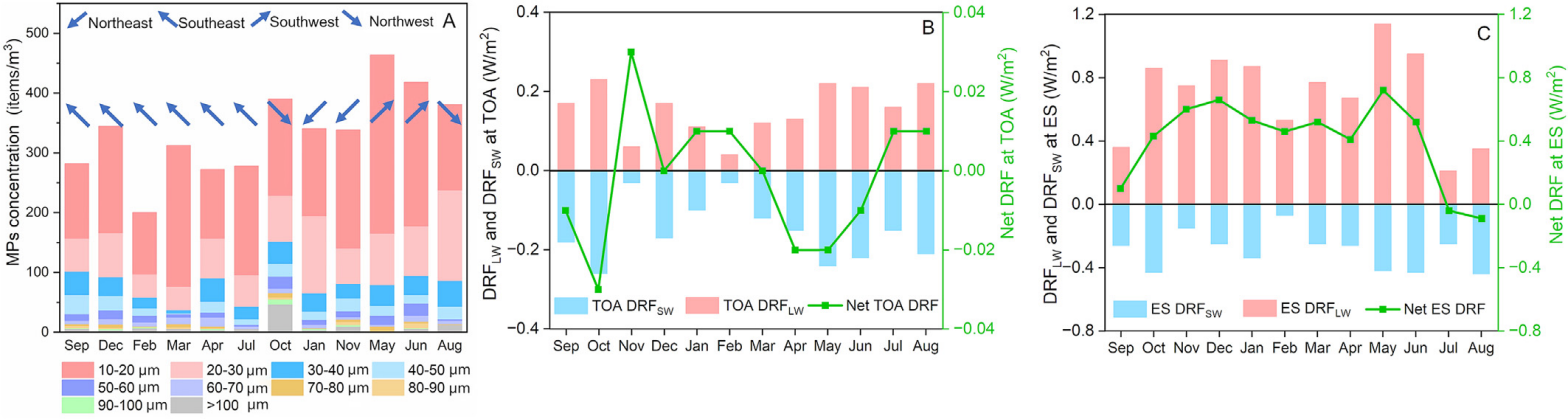
The monthly average concentration of AMPs in Tianjin from September 2021 to August 2022 (A), and the calculated monthly variation of shortwave DRF (DRF_SW_), longwave DRF (DRF_LW_), and the net DRF at the top of the atmosphere (TOA) (B) or Earth's surface (ES) (C). The prevailing wind direction during the sampling period was represented by blue arrows.

The detected concentrations of AMPs in Tianjin, China ranged from 200.0 items/m^3^ to 463.9 items/m^3^, with the quantity distribution of AMPs increasing with a decrease in AMP size (Fig. 1A). The reported concentrations of AMPs vary depending on the resolution of the detecting methods [[25], [26], [27]]. In this study, AMPs between 10 and 20 μm accounted for approximately 50% of all detected AMPs, with concentrations varying from 102.8 to 298.6 items/m^3^ (Fig. 1A). This is in the same order of magnitude as the AMP concentrations reported in London for the same particle size range [26].

Meteorological factors can affect the distribution of AMPs. The influence of wind, rainfall, pressure, temperature, and relative humidity on the concentration of AMPs was analyzed, and a significant negative correlation (*P* < 0.05) between the AMP concentration and frequency of southeast wind was presented (Fig. S4). In coastal cities, clean sea breezes can dilute the AMPs in the urban atmosphere [28]. The Bohai Sea lies to the southeast of Tianjin, and the AMP concentration over the ocean is commonly lower than that over the land [25], which explains why the average concentration of AMPs is low during the prevalence of southeastern winds (Fig. 1A).

### 3.2 DRF of AMPs in Tianjin

The shortwave DRFs (DRF_SW_) of AMPs were calculated by the SBDART model as −0.03 to −0.21 W/m^2^ at TOA and −0.07 to −0.44 W/m^2^ at ES in Tianjin, China (Fig. 1B and C). Meanwhile, the longwave DRFs (DRF_LW_) of AMPs were calculated as 0.03–0.18 and 0.16–0.99 W/m^2^ at TOA and ES, respectively (Fig. 1B and C). Negative DRF_SW_ and positive DRF_LW_ suggest that AMPs led to a cooling effect in the shortwave band and a warming effect in the longwave band. This can be explained by the optical properties of polymers, which scatter radiation of shortwave and absorb radiation of longwave [11].

Although solar radiation is primarily shortwave, once the ES absorbs the shortwave radiation, it emits longwave radiation. Consequently, when calculating net radiative forcing, it is essential to combine the shortwave radiative forcing with the longwave radiative forcing. This is a commonly used method for calculating the net radiative forcing of aerosols, especially for aerosols that can absorb longwave radiation [1]. The annual net DRF of AMPs with different directions at ES and TOA, i.e. 0.402 ± 0.256 W/m^2^ at ES and −0.002 ± 0.016 W/m^2^ at TOA were obtained. The net DRF at ES was positive in most months (0.10–0.72 W/m^2^) except for that in July and August (−0.04 and −0.09 W/m^2^, respectively) (Fig. 1C). In contrast, the net DRF at TOA demonstrated greater uncertainty in the direction of DRFs, with half of the months being negative (−0.01 to −0.03 W/m^2^) and half being positive (0.01–0.03 W/m^2^) (Fig. 1B). AMPs can scatter shortwave radiation (RAD_SW_) of solar radiation [11], thus causing a negative DRF_SW_ at ES. The absorption of longwave radiation (RAD_LW_) of the ground by AMPs can cause positive radiative forcing at ES [11]. The DRF_LW_ is larger than the DRF_SW_ at ES, thus causing a positive net DRF. With the increased altitude, the DRF_LW_ changes more rapidly since longwave radiation (RAD_LW_) is sensitive to water vapor and decreased temperature [29]. As a whole, DRF_SW_ and DRF_LW_ of similar magnitude were obtained at TOA, resulting in a small net DRF with uncertain directions (Fig. 1B).

**Fig. 2:**
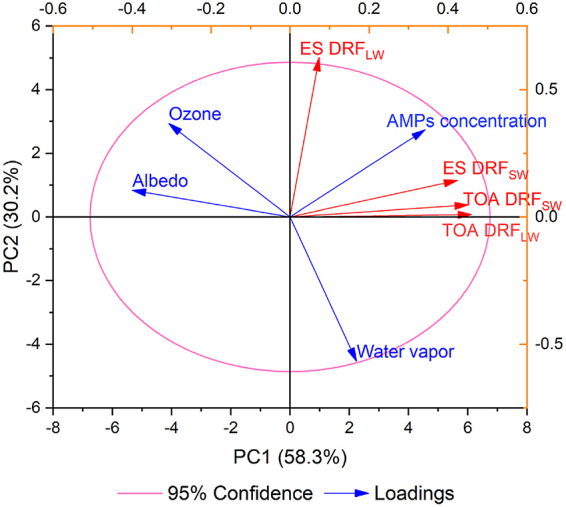
Principal component analysis (PCA) biplot of DRFs of AMPs and AMPs concentration, as well as the environmental parameters of column ozone, water vapor, and surface albedo (detailed values shown in Table S2). ES DRF_LW_, ES DRF_SW_, TOA DRF_LW_, and TOA DRF_SW_ represent the longwave DRF at Earth's surface, the shortwave DRF at Earth's surface, the longwave DRF at the top of the atmosphere, and the shortwave DRF at the top of the atmosphere, respectively. The percentages of PC1 and PC2 indicate the total variance explained by Principal Components 1 and 2. The total variance in the data matrix represented by PC1 and PC2 suggests that these two principal components can effectively capture the complexity and structure of the original dataset in the process of dimensionality reduction.

Columnar ozone, water vapor, and surface albedo can affect the radiative fluxes and are important in estimating aerosol DRF [1]. The influence of these parameters on the absolute value of the DRF of AMPs was analyzed (Fig. 2). Principal components PC1 and PC2 explained 88.5% of the total variance in the data matrix. All DRFs were significantly positively correlated with AMP concentration (*P* < 0.05, Fig. 2). Therefore, the lowest DRF_SW_ at TOA and ES (−0.03 and −0.07 W/m^2^, respectively, Fig. 1B and C) and the lowest DRF_LW_ at TOA (0.04 W/m^2^, Fig. 1B) were all observed in February in Tianjin, which showed the lowest detected AMPs concentration (Fig. 1A). A negative correlation was observed between DRF_LW_ at ES and water vapor amount (Fig. 2). Although DRF_LW_ at ES was not significantly correlated with water vapor amount (*P* > 0.05), it is worth noting that months with the highest water vapor (2.532–3.818 cm in July to September, Table S2) showed the lowest ES DRF_LW_ (0.21–0.36 W/m^2^, Fig. 1C) in the whole year, while detected AMPs concentrations of these months were not the lowest (Fig. 1A). This may be because water vapor near the ground can strongly absorb the RAD_LW_ of the ground [30], thus affecting the ES DRF_LW_. DRFs, except for DRF_LW_ at ES, were significantly negatively correlated with surface albedo (*P* < 0.05 for TOA DRF_SW_, *P* < 0.01 for TOA DRF_LW_ and ES DRF_SW_, Fig. 2). For aerosols that scatter the solar RAD_SW_, more positive DRF_SW_ will be obtained with the increase of surface albedo [31]. For airborne materials that can absorb RAD_LW_ from the ground, such as greenhouse gases, the DRF_LW_ will be more negative under higher surface albedo [32]. MPs are efficient at scattering RAD_SW_ and absorbing RAD_LW_ [11]. Therefore, the lowest DRF_SW_ at TOA (−0.03 W/m^2^, Fig. 1B), the second-to-last lowest DRF_LW_ at TOA (0.06 W/m^2^, Fig. 1B), and DRF_SW_ at ES (−0.15 W/m^2^, Fig. 1C) in Tianjin were all observed in November, which showed the highest surface albedo (0.213, Table S2). Ozone can absorb both RAD_SW_ and RAD_LW_ [33]. However, there was no significant correlation (*P* > 0.05) between DRFs of AMPs and columnar ozone presented, indicating that ozone has a limited effect on DRFs of AMPs.

### 3.3 The DRF of AMPs under different surface albedo

In November, with the highest surface albedo (0.213) due to snowfall events (Table S1), low DRF_SW_ and DRF_LW_ (Fig. 1B and C) were observed, although the detected AMP concentration in November was higher than the annual average concentration (Fig. 1A). This indicates that surface albedo significantly affects the DRF of AMPs. Since a negative DRF_SW_ and a positive DRF_LW_ were found in Tianjin (Fig. 1B and C), and a significant negative correlation was also found between the surface albedo and the absolute values of DRFs of AMPs (Fig. 2), it can be inferred that an increased surface albedo will lead to a positive shift in DRF_SW_ while a negative shift in DRF_LW_, which leads to a more uncertain net DRF at both TOA and ES.

**Fig. 3:**
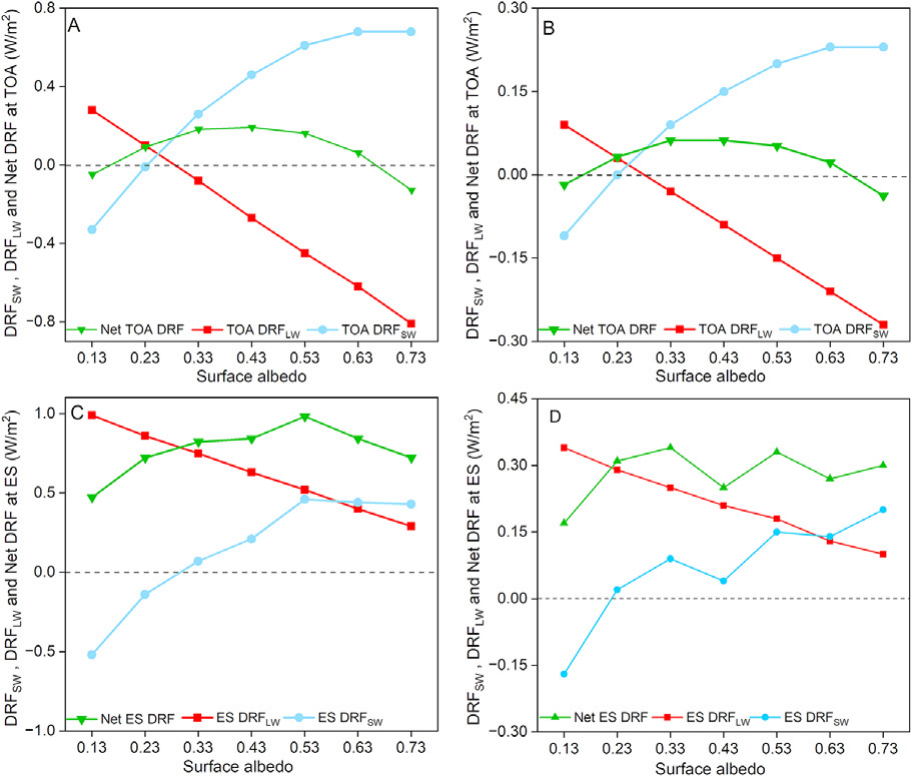
The calculated DRFs at TOA (A,B) and ES (C,D) as a function of surface albedos, based on AMP concentrations of 600 items/m^3^ (A,C) and 200 items/m^3^ (B,D), respectively. The values of ozone and water vapor used in the calculation are shown in Table S4.

To further describe the effect of surface albedo on the DRF of AMPs, the DRFs calculated based on two AMP concentrations (200 and 600 items/m^3^) as a function of the surface albedo at a range of 0.13–0.73 were provided. The lowest and highest monthly AMP concentration levels in Tianjin were 200 and 600 items/m^3^, respectively (Table S4), the lowest and the highest surface albedo in Tianjin during sampling time were 0.13 in October and 0.73 in November when the ground was covered by snow. The AMP concentration near the ground surface is higher; therefore, compared to DRF_SW_ at TOA (Fig. 3A and B), greater fluctuations in DRF_SW_ at ES (Fig. 3C and D) with changes of surface albedo were observed. Overall, the concentration of AMPs affects the magnitudes of net DRF (Fig. S5) but does not affect its direction (Fig. 3). Positive net DRF at ES was observed at all different surface albedos (Fig. 3C and D). The net DRF of AMPs at TOA became increasingly positive in the surface albedo range of 0.13–0.43 (Fig. 3A and B), while decreased at surface albedo of 0.53 compared to that at 0.43, although the net DRF was still positive. When the surface albedo was 0.73, the net DRF at TOA became negative again (Fig. 3A and B). On surfaces with high albedo, e.g., the Antarctic ice sheet, negative net DRF induced by dust has been reported at both TOA and ES [34]. For aerosols that scatter shortwave solar radiation and absorb longwave radiation emitted from the ground, opposite radiative forcings in the shortwave and longwave band will lead to uncertainty in the direction of the DRF at different surface albedo [35]. As the surface albedo increases, the RAD_SW_ reflected by the ground increases. The repeated reflection of RAD_SW_ between the surface and AMPs leads to an extra absorption of RAD_SW_ by atmospheric gases and surface, which can be described as a polynomial function related to the surface albedo [36]. Thus, in contrast to the linear relationship observed between DRF_LW_ and surface albedo, the relationship between DRF_SW_ and surface albedo did not demonstrate a linear trend (Fig. 3A–D). At high surface albedos, the DRFsw at TOA changes slowly with the increase of surface albedo, which results in the net DRF having the same direction as DRF_LW_ at TOA.

The variation of the net DRF at TOA at different surface albedos was calculated as −0.09 to 0.13 W/m^2^ in this study (Fig. 3A and B). The surface albedo exhibits a greater influence on the DRF of AMPs than the AMP concentration (Fig. 3A and B). Thus, it can be inferred that for regions with a surface albedo of ∼0.1, such as water bodies (rivers, lakes, and oceans) [37] and forests [38], the net DRF at TOA is expected to be negative (Fig. 3A and B). For regions with a surface albedo of ∼0.15, such as urban and cropland areas [38], the net DRF at TOA may be very small (Fig. 3A and B) since DRF_LW_ and DRF_SW_ almost cancel each other out. In grass and bare soil lands, net DRF at TOA tends to be positive because of the relatively high surface albedo (approximately 0.2–0.23 [38,39]). For regions covered by snow and ice, which show the highest surface albedo (approximately 0.8) [40] among different land cover types, the net DRF of AMPs at TOA is more likely to be negative (Fig. 3A and B). The radiative forcing at ES primarily reflects changes in the surface radiation balance, which can be used to assess the impact of aerosol radiative forcing on the hydrological cycle, soil moisture and aridity, and vegetation physiology [14]. Compared to the net DRF at ES, which represents the disturbance of aerosol to the radiation balance at ES, the net DRF at TOA represents the disturbance of aerosol to the radiation balance of the Earth-atmosphere system [41]. Thus, net DRF at TOA can be used to evaluate the climate change caused by AMPs [42]. That is, a positive net DRF at TOA indicates a warming effect in the atmosphere, while a negative net DRF indicates a cooling effect. Considering that oceans account for approximately 70% of the Earth's surface, AMPs are expected to cause a cooling effect in most regions on the planet. For terrestrial environments, regions of grasslands and bare soil, where AMPs are prone to cause a warming effect, account for 43% of the total land area (Table S5). In comparison, regions of the forests, rivers, lakes, and land covered by snow and ice, where AMPs are prone to cause a cooling effect, account for 41% of the total land area (Table S5).

## 4 Conclusion

The monthly AMP concentration in Tianjin from September 2021 to August 2022 was detected, and the DRF of AMPs was calculated. The concentration of AMPs varied from 200.0 to 463.9 items/m^3^, showing a significant negative correlation with the frequency of southeast wind, suggesting that clean air from the ocean can diffuse AMPs in urban areas. It was found that the surface albedo has a great influence on the direction of the DRF of AMPs at TOA, which determines the radiative effects caused by AMPs. Compared to the concentration of AMPs, the direction of the DRF of AMPs is more dependent on the surface albedo. It is expected that when the land cover is urban or cropland (with a surface albedo of ∼0.15), the radiative impact of AMPs in the atmosphere is weak. For the surface with low albedo, e.g., water bodies and forests (with a surface albedo of ∼0.1), and land covered by snow or ice (with a surface albedo of ∼0.8), AMPs tend to cause a cooling effect in the atmosphere. On the contrary, over grassland and bare soil with higher surface albedo (0.2–0.3), AMPs tend to cause a warming effect in the atmosphere. The results indicate that AMPs in different regions may cause different radiative effects, which are largely related to the characteristics of the underlying surface. Future studies should focus on actual measurements of the vertical distribution of AMPs and the refractive index of pigmented plastics.

## CRediT authorship contribution statement

**Hanling Yang:** Writing – original draft, Methodology, Investigation, Formal analysis, Data curation. **Yining Xue:** Methodology, Investigation. **Xiaoyu Sha:** Methodology, Data curation. **Jintao Yang:** Methodology, Data curation. **Xinling Wang:** Methodology, Investigation. **Balt Suvdantsetseg:** Validation, Investigation. **Keisuke Kuroda:** Validation, Investigation. **Jian Pu:** Validation, Investigation. **Lei Wang:** Writing – original draft, Supervision, Conceptualization.

## Declaration of competing interest

The authors declare that they have no known competing financial interests or personal relationships that could have appeared to influence the work reported in this paper.
