# Supplementary material for: Influence of regional environmental variables on the radiative forcing of atmospheric microplastics
Source: Eco Environ Health. 2024 Dec 9;4(1):100128. doi: 10.1016/j.eehl.2024.11.002 (PMC11833332; doi:10.1016/j.eehl.2024.11.002)
Supplement: Multimedia component 1 [file mmc1.docx]

***Supplementary Material
For***

**Influence of regional environmental variables on the radiative forcing of atmospheric microplastics**

*Hanling Yang^a^, Yining Xue^a^, Xiaoyu Sha^a^, Jintao Yang^a^, Xinling Wang^a^, Balt Suvdantsetseg^b^, Keisuke Kuroda^c^, Jian Pu^d,e^, Lei Wang^a,^**

^a^ MOE Key Laboratory of Pollution Processes and Environmental Criteria/Tianjin Key Laboratory of Environmental Remediation and Pollution Control, College of Environmental Science and Engineering, Nankai University, Tianjin 300350, China

^b^ Department of Administration and International cooperation, Mongolian Academy of Sciences, Ulaanbaatar, Mongolia

^c^ Department of Environmental and Civil Engineering, Toyama Prefectural University, Kurokawa 5180, Imizu City, Toyama 939-0398, Japan

^d^ Institute for the Advanced Study of Sustainability, United Nations University, Jingumae 5-53-70, Shibuya-ku, Tokyo 150-8925, Japan

^e^ Institute for Future Initiatives, The University of Tokyo, Tokyo 113-0033, Japan

* Corresponding author, E-mail address: wang2007@nankai.edu.cn

*Number of Pages*: *15
Number of Texts*: *3
Number of Figures*: *5
Number of Tables*: *5***Text S1.** Preservation and pre-treatment of samples.

After sampling, filters were carefully placed into clean aluminum foil, and transferred to the laboratory immediately. Each filter membrane was uniformly cut into four petals. For the two samples collected in the same month, 1/4 of the filter was taken from each sample and then subjected to ultrasonic oscillation. AMPs on filters were digested and floated by 30% H_2_O_2_ and 52% ZnCl_2_ solution for 12 h, respectively. After the flotation process, the upper layer of ZnCl_2_ solution was filtered through a mental filer (pore size of 10 μm). Then, the mental filter was transferred into a 50 mL glass tube with 20 mL of HPLC-grade ethanol and sonicated at 40,000 Hz for 1 h. Then, the mental filter was removed by stainless steel tweezers, and the ethanol solution was concentrated into 1 mL with nitrogen. The concentrated solution was dropped on a high reflection glass window for LDIR detection after the ethanol volatilized.

**Text S2.** Details of observing the color of AMPs.

From each 1 mL ethanol solution prepared for LDIR detection (Text S1), 100 mL of the solution was taken onto a glass fiber membrane (GF/F Whatman) with a diameter of 47 mm and a pore size of 0.7 μm. The solution was subjected to vacuum filtration, and the filter membrane was devided into eight equal fields of view. Using a stereo microscope (SZN71TR-B4, 0.65X-4.5X), particles within each field of view were inspected. The color of each MPs particle was documented until a total of 100 MPs were identified. Finally, the percentage of white and transparent MPs among the identified particles was calculated. The visual identification of MPs follows the principles that plastics must not contain any biogenic (cellular or organic) structures, and fragments should exhibit a relatively consistent color and the same degree of transparency or clarity^1^.

**Text S3.** The detailed quality assurance for the AMPs detection.

Glass microfiber filters, stainless tweezers and glass tubes were wrapped in aluminum then baked at 500 ℃ in a muffle furnace for 4 h. Mental filters were rinsed twice with filtered pure water before use. Procedure of the sample pretreatment was conducted in the fume hood, and plastic products were avoided except for the polypropylene pipette tips, which were also rinsed with filtered pure water before use. The procedure blanks were subjected to the same treatment as the suspended particulate matters, and no MP background was detected by LDIR.


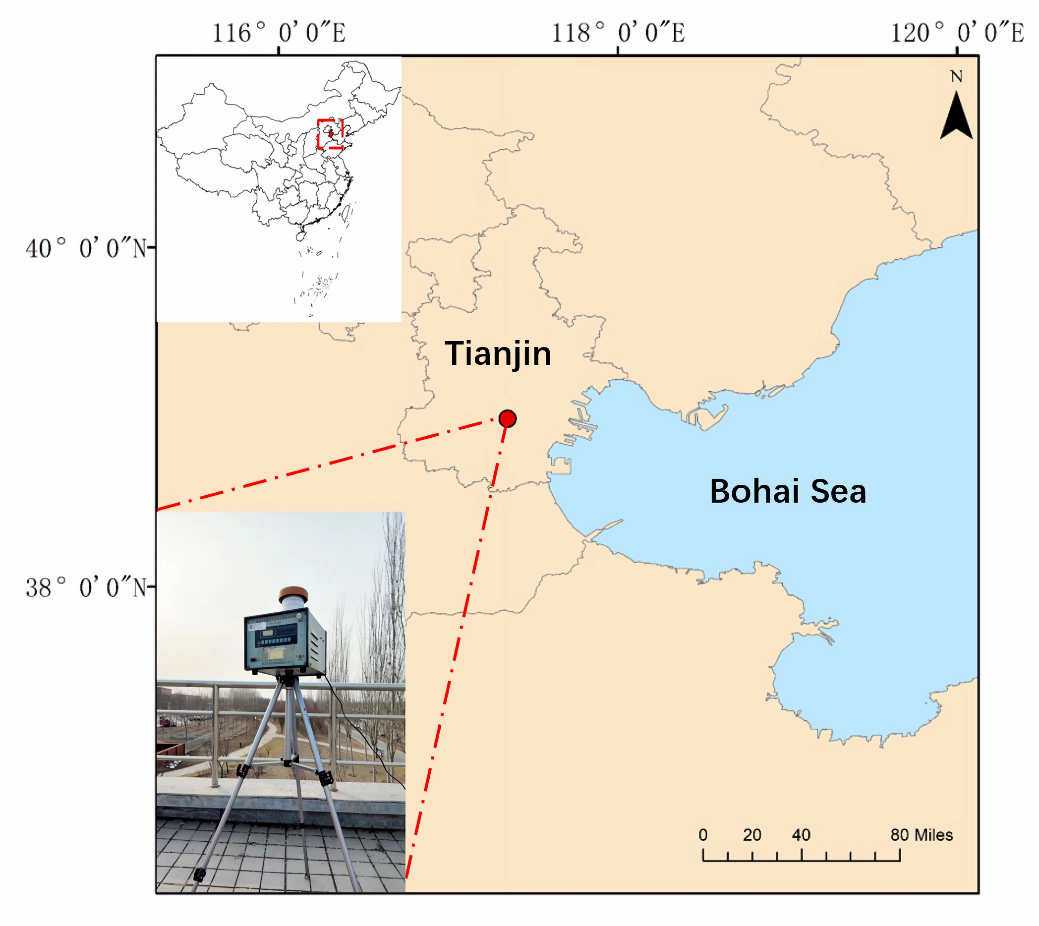


**Figure S1.** Location of the sampling site of suspended particulate matters.


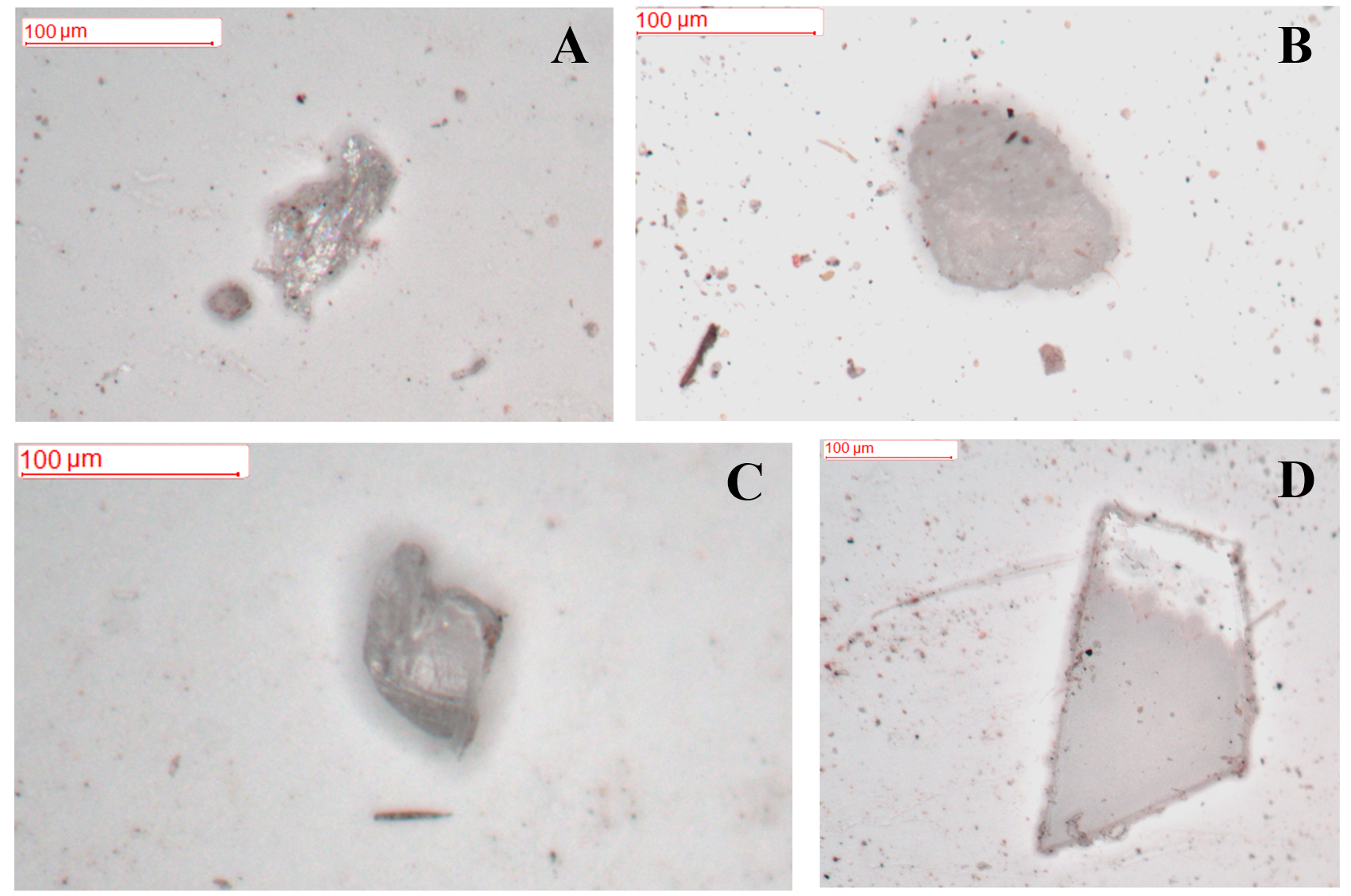


**Figure S2.** The color of 100 randomly selected AMPs particles, with 75% of them being white and transparent (the scale is set at 100 micrometers). Details of observing the color of AMPs was shown in Text S2.





**Figure S3.** Size groups of AMPs in Tianjin, based on all AMPs detected by LDIR.


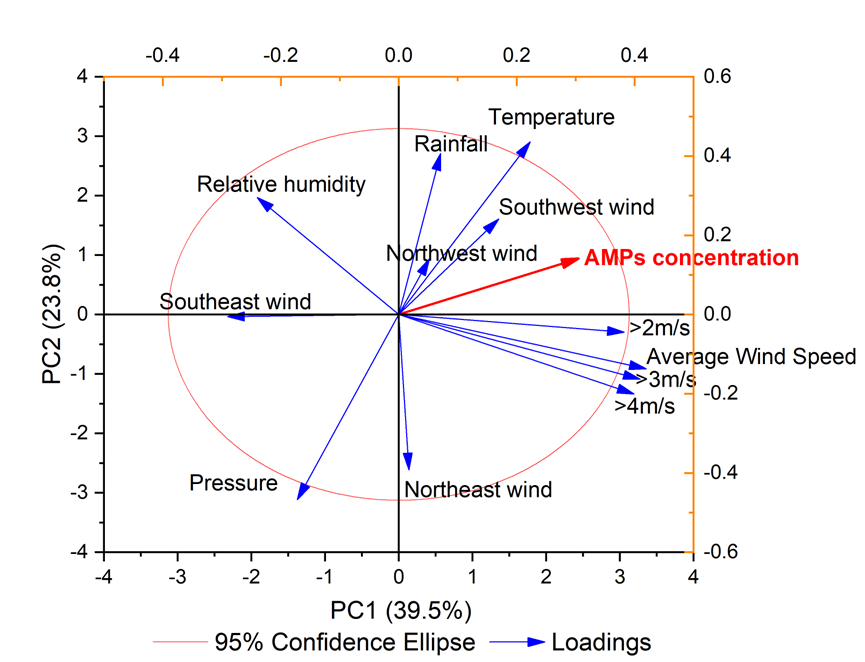


**Figure S4.** Principal component analysis (PCA) biplot of the AMPs concentration, average wind speed, frequency of wind speeds exceeding 2 m/s, 3 m/s and 4 m/s, frequency of different wind directions, pressure, relative humidity, temperature and rainfall.

**
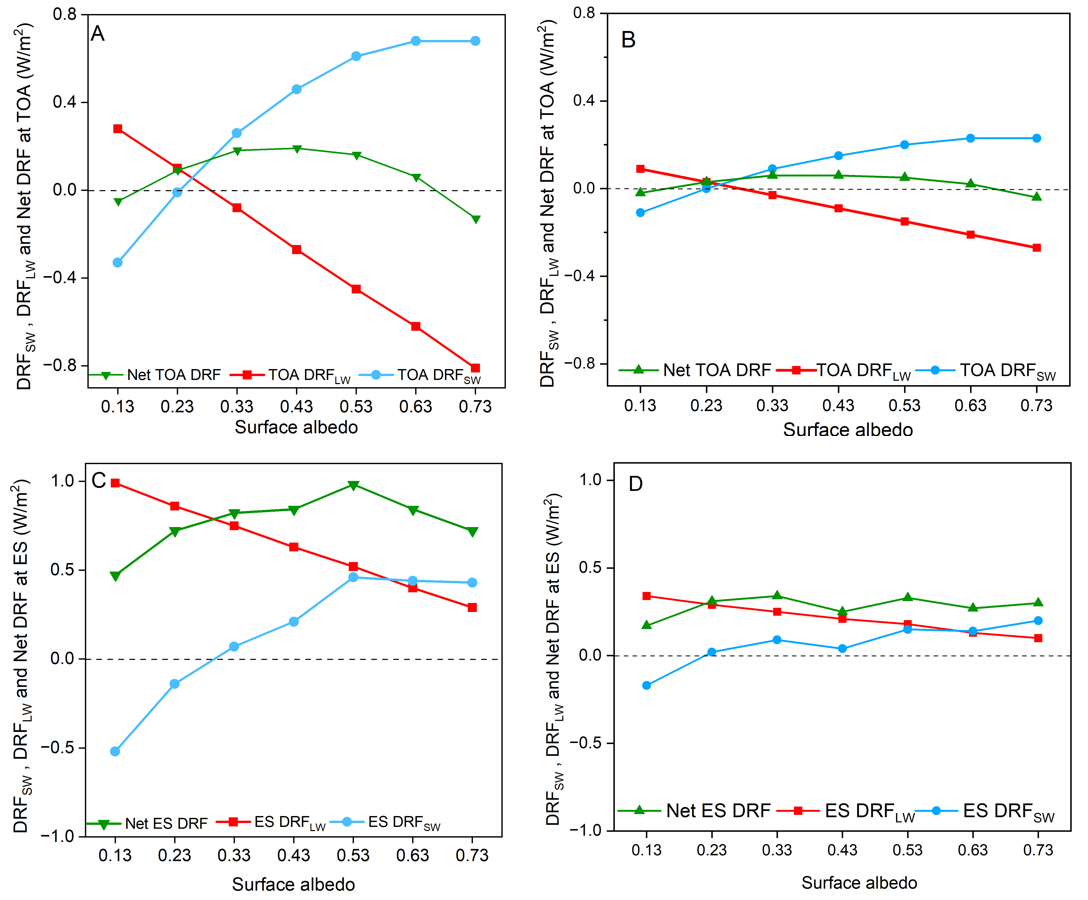
**

**Figure S5.** The calculated DRFs at TOA (A,B) and ES (C,D) as a function of surface albedos, based on AMPs concentrations of 600 items/m^3^ (A,C) and 200 items/m^3^ (B,D).

**Table S1.** Meteorological data of Tianjin during the sampling period

| Sampling time | Sep | Oct | Nov | Dec | Jan | Feb | Mar | Apr | May | Jun | Jul | Aug |
| --- | --- | --- | --- | --- | --- | --- | --- | --- | --- | --- | --- | --- |
| Average Wind speed (m/s) | 1.95 | 3.89 | 1.85 | 1.81 | 2.06 | 2.86 | 2.9 | 3.14 | 4.34 | 3.19 | 2.35 | 3.20 |
| Frequency of wind speed > 2 m/s | 0.42 | 0.90 | 0.40 | 0.42 | 0.44 | 0.69 | 0.73 | 0.71 | 0.85 | 0.73 | 0.56 | 1.00 |
| Frequency of wind speed > 3 m/s | 0.00 | 0.69 | 0.00 | 0.00 | 0.17 | 0.33 | 0.38 | 0.4 | 0.69 | 0.52 | 0.27 | 0.40 |
| Frequency of wind speed > 4 m/s | 0.00 | 0.54 | 0.00 | 0.00 | 0.02 | 0.23 | 0.19 | 0.23 | 0.56 | 0.31 | 0.08 | 0.17 |
| Northeast wind frequency | 0.31 | 0.75 | 0.00 | 0.06 | 0.35 | 0.31 | 0.35 | 0.17 | 0.02 | 0.13 | 0.15 | 0.00 |
| Southeast wind frequency | 0.31 | 0.02 | 0.15 | 0.58 | 0.23 | 0.38 | 0.48 | 0.44 | 0.04 | 0.23 | 0.58 | 0.13 |
| Southwest wind frequency | 0.19 | 0.15 | 0.83 | 0.19 | 0.15 | 0.08 | 0.10 | 0.33 | 0.90 | 0.31 | 0.25 | 0.38 |
| Northwest wind frequency | 0.19 | 0.08 | 0.02 | 0.17 | 0.27 | 0.23 | 0.06 | 0.06 | 0.04 | 0.33 | 0.02 | 0.50 |
| Snowfall amount (mm) | 0.00 | 0.00 | 27.1 | 0.27 | 5.78 | 5.50 | 4.81 | 0.00 | 0.00 | 0.00 | 0.00 | 0.00 |
| Rainfall (mm) | 121 | 83.5 | 55.5 | 0.55 | 4.18 | 7.42 | 18.4 | 10.6 | 24.7 | 54.0 | 157 | 192 |
| Pressure (hPa) | 1013 | 1029 | 1019 | 1023 | 1029 | 1035 | 1017 | 1018 | 1006 | 999.0 | 1001 | 1003 |
| Relative humidity (%) | 87.0 | 66.4 | 85.8 | 75.4 | 51.5 | 44.4 | 59.0 | 47.0 | 37.5 | 45.0 | 75.4 | 71.5 |
| Temperature (℃) | 22.7 | 11.0 | 9.27 | 3.15 | -3.14 | -2.94 | 3.92 | 16.4 | 23.9 | 30.2 | 29.2 | 30.5 |

**Table S2.** The monthly varied columnar ozone, surface albedo and water vapor amount in Tianjin, ^a^ during September 2021 to August 2022

| Sampling time | Sep | Oct | Nov | Dec | Jan | Feb | Mar | Apr | May | Jun | Jul | Aug |
| --- | --- | --- | --- | --- | --- | --- | --- | --- | --- | --- | --- | --- |
| Columnar ozone (DU) | 293.6 | 287.2 | 326.2 | 331.3 | 359.1 | 383.5 | 350.9 | 347.0 | 354.1 | 332.1 | 307.8 | 292.8 |
| Surface albedo | 0.140 | 0.134 | 0.213 | 0.151 | 0.182 | 0.198 | 0.168 | 0.151 | 0.151 | 0.151 | 0.155 | 0.151 |
| Water vapor (cm) | 2.532 | 0.979 | 0.617 | 0.363 | 0.362 | 0.253 | 0.498 | 0.553 | 0.357 | 0.797 | 3.818 | 3.303 |

^a^ Columnar ozone was obtained from Ozone Monitoring Instrument, surface albedo was obtained from ERA5-LAND and columnar water vapor was obtained from AERONET (Xianghe and Beijing stations). For water vapor, the data in principle came from the observation station closest to the sampling location. When data from the nearest station was missing, the data from another neighboring observatory was used as a supplement.

| Wave length (μm) |  |  |  |  | AMPs concentration (n/m^3^) | | | | | | | | | | | |
| --- | --- | --- | --- | --- | --- | --- | --- | --- | --- | --- | --- | --- | --- | --- | --- | --- |
|  |  | 200 | | |  | 300 | | |  | 400 | | |  | 500 | | |
|  |  | ASY | SSA | AOD |  | ASY | SSA | AOD |  | ASY | SSA | AOD |  | ASY | SSA | AOD |
| 0.50 |  | 0.805 | 0.9989 | 0.0053 |  | 0.805 | 0.9989 | 0.0079 |  | 0.805 | 0.9989 | 0.0105 |  | 0.805 | 0.9989 | 0.0131 |
| 1.00 |  | 0.808 | 0.9957 | 0.0053 |  | 0.808 | 0.9957 | 0.0079 |  | 0.808 | 0.9957 | 0.0106 |  | 0.808 | 0.9957 | 0.0132 |
| 2.00 |  | 0.811 | 0.9633 | 0.0054 |  | 0.811 | 0.9633 | 0.0081 |  | 0.811 | 0.9633 | 0.0107 |  | 0.811 | 0.9633 | 0.0134 |
| 4.10 |  | 0.850 | 0.7902 | 0.0055 |  | 0.850 | 0.7902 | 0.0082 |  | 0.850 | 0.7902 | 0.0110 |  | 0.850 | 0.7902 | 0.0137 |
| 8.00 |  | 0.893 | 0.6262 | 0.0056 |  | 0.893 | 0.6262 | 0.0085 |  | 0.893 | 0.6262 | 0.0113 |  | 0.893 | 0.6262 | 0.0141 |
| 16.5 |  | 0.876 | 0.6029 | 0.0059 |  | 0.876 | 0.6029 | 0.0089 |  | 0.876 | 0.6029 | 0.0119 |  | 0.876 | 0.6029 | 0.0149 |
| 32.5 |  | 0.782 | 0.7077 | 0.0065 |  | 0.782 | 0.7077 | 0.0097 |  | 0.782 | 0.7077 | 0.0129 |  | 0.782 | 0.7077 | 0.0162 |

**Table S3.** The optical parameters^a^ of AMPs in the boundary layer calculated based on different AMPs concentrations^b^

^a^ ASY、SSA and AOD were calculated based on the source data ([https://www.nature.com/articles/s41586-021-03864-x#MOESM10/](https://www.nature.com/articles/s41586-021-03864-x#MOESM10/)) of Revell et al.^2^

^b^ Referring to the AMPs concentrations on the Earth surface obtained from atmospheric suspended particulate samples.

**Table S4.** The AMPs concentrations^a^, columnar ozone^b^ and water vapor amount^c^, were used in DRFs calculation ^d^

| The highest level of AMPs concentration (n/m^3^) | The lowest level of AMPs concentration (n/m^3^) | Columnar ozone  (DU) | Water vapor  (cm) |
| --- | --- | --- | --- |
| 600 | 200 | 331.0 | 1.203 |

^a^ Monthly AMPs concentrations in Tianjin ranged from 250 to 579.9 n/m^3^. The concentration of 200 and 600 n/m^3^ represents the highest and lowest AMPs level, respectively.

^b,c^ Both the columnar ozone of 331.0 DU and the water vapor of 1.203 cm are annual averages, derived from monthly data in Tianjin (Table S2).

^d^ The calculated DRFs were shown in Figure 5.

**Table S5.** Proportion of different land cover types on the Earth's surface^a^ and the corresponding surface albedos

| Land cover types | Urban | Snow and ice | Bare soil | Crop land | Forest | Grass land | Water bodies |
| --- | --- | --- | --- | --- | --- | --- | --- |
| Land cover percentage (%) | 0.2 | 9.7 | 13.3 | 15.7 | 29.4 | 30 | 2 |
| Surface albedo | 0.16 | 0.8 | 0.23 | 0.15 | 0.11 | 0.20 | 0.1 |

^a^ Data of land cover types were derived from the World Agriculture and Food Organization (https://data.apps.fao.org/).

**Reference**

[1] Y. Zhang, S. Kang, S. Allen, D. Allen, T. Gao, S. Mika, Atmospheric microplastics: A review on current status and perspectives, Earth-Science Reviews. 203 (2020) 103118.

[2] L.E. Revell, P. Kuma, E.C. Le Ru, W.R.C. Somerville, S. Gaw, Direct radiative effects of airborne microplastics, Nature. 598 (2021) 462–467.
